# Significant Growth Inhibition of Canine Mammary Carcinoma Xenografts following Treatment with Oncolytic Vaccinia Virus GLV-1h68

**DOI:** 10.1155/2010/736907

**Published:** 2010-06-23

**Authors:** Ivaylo Gentschev, Klaas Ehrig, Ulrike Donat, Michael Hess, Stephan Rudolph, Nanhai Chen, Yong A. Yu, Qian Zhang, Jörn Bullerdiek, Ingo Nolte, Jochen Stritzker, Aladar A. Szalay

**Affiliations:** ^1^Genelux Corporation, San Diego Science Center, San Diego, CA 92109, USA; ^2^Department of Biochemistry, University of Wuerzburg, 97074 Wuerzburg, Germany; ^3^Center for Human Genetics, University of Bremen, 28359 Bremen, Germany; ^4^Small Animal Clinic, University of Veterinary Medicine, Bischofsholer Damm 15, 30173 Hannover, Germany; ^5^Rudolf Virchow Center for Experimental Biomedicine, University of Wuerzburg, 97078 Wuerzburg, Germany; ^6^Institute for Molecular Infection Biology, University of Wuerzburg, 97078 Wuerzburg, Germany; ^7^Department of Radiation Oncology, Moores Cancer Center, University of California, San Diego, 3855 Health Sciences Drive 0843, La Jolla, CA 92093-0843, USA

## Abstract

Canine mammary carcinoma is a highly metastatic tumor that is poorly responsive to available treatment. Therefore, there is an urgent need to identify novel agents for therapy of this disease. Recently, we reported that the oncolytic vaccinia virus GLV-1h68 could be a useful tool for therapy of canine mammary adenoma *in vivo*. In this study we analyzed the therapeutic effect of GLV-1h68 against canine mammary carcinoma. Cell culture data demonstrated that GLV-1h68 efficiently infected and destroyed cells of the mammary carcinoma cell line MTH52c. Furthermore, after systemic administration, this attenuated vaccinia virus strain primarily replicated in canine tumor xenografts in nude mice. Finally, infection with GLV-1h68 led to strong inflammatory and oncolytic effects resulting in significant growth inhibition of the tumors. In summary, the data showed that the GLV-1h68 virus strain has promising potential for effective treatment of canine mammary carcinoma.

## 1. Introduction

Malignant tumors of the mammary glands are among the most frequently observed tumors in female dogs [1–3]. Despite the success in diagnosis and treatment of mammary cancer, this disease entity remains one of the leading causes of cancer-related death in female dogs. Therefore, there is an urgent need to identify novel agents for therapy and diagnosis of mammary cancer. Among the most promising new therapeutic candidates are oncolytic viruses, which can target tumor tissue and specifically eradicate the cancer cells. This concept was already confirmed in human tumor xenograft treatment by the use of several viruses [4–9]. 

In the present study, we tested the recombinant oncolytic vaccinia virus GLV-1h68 as a therapeutic agent against canine mammary carcinoma. The GLV-1h68 virus strain was engineered by inserting expression cassettes encoding a *Renilla* luciferase-green fluorescent protein (GFP) fusion protein, *β*-galactosidase, and *β*-glucuronidase into the genome of the wild-type strain LIVP [10]. In nude mouse models, GLV-1h68 is highly attenuated compared to the wild-type strain [10]. We have already demonstrated that the injection of GLV-1h68 leads to regression and elimination of different tumor xenografts in nude mice [10–15]. More recently, we reported that GLV-1h68 could be a useful tool for therapy of canine mammary adenoma [12].

Here we describe that the GLV-1h68 virus successfully infected, replicated, and lysed canine mammary carcinoma MTH52c cells in cell culture. In addition, GLV-1h68 can efficiently prevent cancer growth in female nude mice with tumors derived from MTH52c cells. Finally, the localization and effects of GLV-1h68 in the primary tumor were analyzed by immunohistochemical studies and by mouse Immune-Related Protein Antigen Profiling.

## 2. Materials and Methods

### 2.1. Cell Culture

African green monkey kidney fibroblasts (CV-1) were obtained from the American Type Culture Collection (ATCC). MTH52c is derived from a malignant small-cell canine carcinoma [16]. 

Cells were cultured in DMEM supplemented with antibiotic solution (100 U/ml penicillin G, 100 units/ml streptomycin) and 10% fetal bovine serum (FBS; Invitrogen GmbH, Karlsruhe, Germany) for CV-1 and 20% FBS for MTH52c at 37°C under 5% CO_2_.

### 2.2. Virus Strain

GLV-1h68 is a genetically stable oncolytic virus strain designed to locate, enter, colonize, and destroy cancer cells without harming healthy tissues or organs [10].

### 2.3. Cell Viability Assay with GLV-1h68

MTH52c cells were seeded in 24-well plates (Nunc, Wiesbaden, Germany). After 24 hours in culture, cells were infected with GLV-1h68 using multiplicities of infection (MOI) of 0.1 and 1.0. Cells were incubated at 37°C for 1 hour, after which the infection medium was removed, and cells were subsequently incubated in fresh growth medium. The amount of viable cells after infection with GLV-1h68 was measured as described previously [12].

### 2.4. Viral Replication

For the viral replication assay, MTH52c cells grown in 24-well plates were infected with GLV-1h68 at an MOI of 0.1. After one hour of incubation at 37°C with gentle agitation every 20 minutes, the infection medium was removed and replaced by a fresh growth medium. After 1, 6, 12, 24, 48, 72, and 96 hours, the cells and supernatants were harvested. Following three freeze-thaw cycles, serial dilutions of the lysates were titered by standard plaque assays on CV-1 cells. All samples were measured in triplicate.

### 2.5. Western Blot Analysis of Virus-Mediated Marker Proteins

Three days prior to infection, MTH52c cells were seeded in 24-well plates (Nunc, Wiesbaden, Germany). If not otherwise indicated, the 90% confluent cell layer was mock-infected or infected with GLV-1h68 at MOIs of 0.1 and 1.0 for 1 hour at 37°C. The virus-containing medium was aspirated and replaced by fresh medium containing 20% FBS. For protein isolation and detection, cells were harvested and resuspended in sodium dodecyl sulfate (SDS) sample buffer at one, 12, 24, 48, 72, and 96 hours post infection (hpi). The protein samples were separated by 10% SDS polyacrylamide gel electrophoresis (PAGE) and subsequently blotted onto a nitrocellulose membrane (Whatman GmbH, Dassel, Germany). The membrane was then incubated with anti-beta-actin mouse monoclonal antibodies (ab6276, Abcam, Cambridge, UK), anti-beta-galactosidase rabbit polyclonal antibodies (A-11132, Molecular Probes, Leiden, Netherlands), or anti-GFP rabbit polyclonal antibodies (sc-8334, Santa Cruz, Heidelberg, Germany), and detection was obtained using horseradish peroxidise-labeled secondary antibodies against mice (ab6728, Abcam, Cambridge, UK) or rabbits (ab6721, Abcam, Cambridge, UK) followed by enhanced chemiluminescence.

### 2.6. Fluorescence Imaging

The GFP signals of virus-infected cells and animals were analyzed with a fluorescence microscope (Leica DM IRB; Wetzlar, Germany) and a fluorescence stereomicroscope (Leica MZ 16 FA; Wetzlar, Germany), respectively. Images were captured with an electronic camera and were processed using META-MORPH (Universal Imaging; Downingtown, PA, USA) and Photoshop 7.0 (Adobe Systems, Mountain View, CA, USA).

### 2.7. Flow Cytometry (FACS) Analysis

MTH52c cells were grown on 24-well plates (Nunc, Wiesbaden, Germany) and infected by GLV-1h68 at an MOI of 0.1 or 1.0, respectively. At various time points, infected and noninfected MTH52c cells were harvested by Trypsin-EDTA treatment (PAA Laboratories GmbH, Pasching, Austria) and resuspended in PBS. For discrimination between viable and dead, MTH52c cells were stained using 2 *μ*l propidium iodide (1mg/ml; Sigma, Taufkirchen, Germany) per 1 ml cell suspension for 5 min at room temperature. A minimum of 2 × 10^5^ cells were then measured using an Epics XL flow cytometer (Beckman Coulter GmbH Krefeld, Germany).

### 2.8. Bioluminescence Imaging

For monitoring studies of the distribution of the GLV-1h68 virus in tumor-bearing mice, animals were analyzed for the presence of virus-dependent luciferase activity. For this purpose, mice were injected intravenously with a mixture of 5 *μ*l of coelenterazine (Sigma, Taufkirchen, Germany; 0.5 *μ*g/*μ*l diluted ethanol solution) and 95 *μ*l of luciferase assay buffer (0.5 M NaCl; 1 mM EDTA; 0.1 M potassium phosphate, pH 7.4). The animals were then anaesthetized with 2.5% Isoflurane (Forene, Abbott, Ludwigshafen, Germany) in a knockout box and were maintained in an anaesthesia module aerated with 1.5% Isoflurane/oxygen. The mice were imaged using the CCD-Camera-based NightOWL LB 981 Imaging System (Berthold Technologies, Bad Wildbad, Germany). Photons were collected for 5 minutes from dorsal views of the animals, and the images were recorded using Image WinLight 32 software (Berthold Technologies, Bad Wildbad, Germany).

### 2.9. GLV-1h68-Mediated Therapy of MTH52c Xenografts

Tumors were generated by 5 × 10^6^ implanting cells in 100 *μ*l PBS subcutaneously into the right hind leg of 6- to 8-week-old female nude mice (NCI/Hsd/Athymic Nude-*F* *o* *x* *n*1^nu^, Harlan Winkelmann GmbH, Borchen, Germany). Tumor growth was monitored 3 times weekly in two dimensions using a digital caliper. Tumor volume was calculated as [(length × width^2^)/2]. On day 12, a single dose of GLV-1h68 virus (5 × 10^6^ plaque forming units [pfu] in 100 *μ*l PBS) was injected into the tail vein (i.v.). The control animals were injected i.v. with PBS only.

The significance of the results was calculated by two-way analysis of variance (ANOVA) using the GraphPad Prism software (San Diego, USA). Results are displayed as means ± s.d. (standard deviation). *P*-values of <.05 were considered significant.

The animals were euthanized by cervical dislocation. All animal experiments were approved by the government of Unterfranken and conducted according to the German animal protection guidelines.

### 2.10. Toxicity Studies

Mice with MTH52c xenograft tumors were developed to assess the biodistribution and toxicity of the GLV-1h68 virus. After virus infection, animals were observed daily for any sign of toxicity, and body weight was checked twice weekly. At day 21 and 42 after injection, viral distribution in animals from each group was analyzed. The tumors and organs were excised, inspected, and homogenized using FastPrep FP120 Cell Disruptor (BIO 101, Qbiogene, Germany) at a speed of 6 for 20 s (three times). After three freeze-thaw cycles, the supernatants were collected by centrifugation at 1000 × g for 5 minutes. The viral titers were then determined in duplicate by standard plaque assays using CV-1 cells.

### 2.11. Histological Analysis of Tumors

For histological studies, tumors were excised and snap-frozen in liquid N_2_, followed by fixation in 4% paraformaldehyde/PBS at pH 7.4 for 16 h at 4°C. Tissue sectioning was performed as described by Weibel et al. [17]. GLV-1h68 was labeled using polyclonal rabbit antivaccinia virus (anti-VACV) antibody (Abcam, Cambridge, UK), which was stained using Cy3-conjugated donkey antirabbit secondary antibodies obtained from Jackson ImmunoResearch (West Grove, PA, USA). Phalloidin-TRITC (Sigma, Taufkirchen, Germany) was used to label actin. 

The fluorescent-labeled preparations were examined using the Leica MZ 16 FA Stereo-Fluorescence microscope equipped with a Leica DC500 Digital Camera. Digital images were processed with Photoshop 7.0 (Adobe Systems, Mountain View, CA, USA) and merged to yield pseudocolored images.

### 2.12. Preparation of Tumor Lysates for Mouse Immune-Related Protein Antigen Profiling

GLV-1h68-infected and noninfected tumors of MTH52c or ZMTH3 xenografted nude mice were used for preparation of tumor lysates at 42 days after virus infection. Tumors were resuspended in 9 volumes (W/V) lysis buffer [50 mM Tris-HCl (pH 7.4), 2 mM EDTA (pH 7.4), 2 mM PMSF and Complete Mini protease inhibitors (Roche, Mannheim, Germany)] and lysed using a FastPrep FP120 Cell Disruptor (BIO 101, Qbiogene, Germany) at a speed of 6.0 for 20 s (three times). Samples were centrifuged at 20,000 g at 4°C for 5 minutes, and supernatants were then analyzed for mouse immune-related protein antigen profiling by Multianalyte Profiles (mouse MAPs; Rules-Based Medicine, Austin, USA) using antibody-linked beads. Results were normalized based on total protein concentration.

## 3. Results

### 3.1. Infection and Replication of GLV-1h68 in the Canine Mammary Cell Line MTH52c

**Figure 1:**
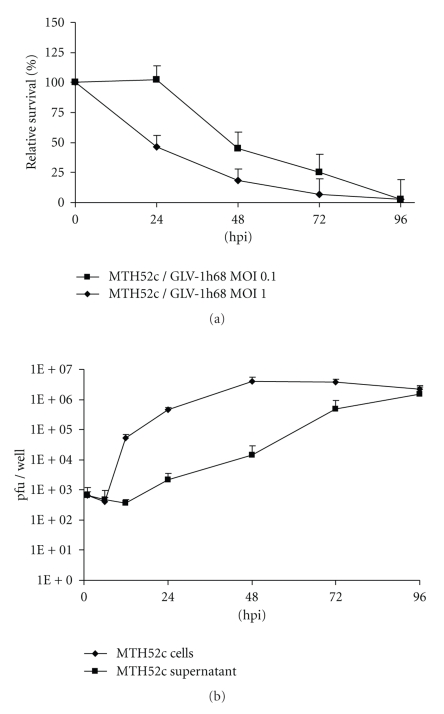
Cytotoxicity (a) and replication (b) of the GLV-1h68 virus in canine mammary MTH52c cells. (a) Viability of MTH52c cells after GLV-1h68 infection using MOIs of 0.1 and 1 was monitored over 96 hours. The amount of viable cells after infection with GLV-1h68 was measured in triplicate. Values are shown as percentages of respective uninfected controls. (b) Viral titer analysis in MTH52c cell culture after infection with GLV-1h68 at an MOI of 0.1. Cells and supernatant of virally treated cells were collected at various times post infection. Viral titers were determined as pfu per well in triplicate by plaque assay in CV-1 cell monolayers. Average plus standard deviations plotted.

In order to test the ability of the GLV-1h68 virus to infect and lyse MTH52c cells in cell culture, we first performed a cell viability assay, as described in Materials and Methods. Ninety-six hours after GLV-1h68 infection at an MOI of 0.1 and 1.0, the MTH52c cells were eradicated, with only 2.4 ± 1.04% and 206 ± 0.68%surviving the treatment, respectively (Figure 1(a)). These results indicate that GLV-1h68 virus infection leads to an efficient eradication of the carcinoma MTH52c cells in culture. 

To determine the replication efficacy of GLV-1h68 in MTH52c cells, we analyzed both the supernatant and the cell-associated virus titers at different times post infection (Figure 1(b)). Whereas the cell-associated virus titer in MTH52c peaked at 48 hours p.i. (3.94 × 10^6^ pfu/well), the maximum yield in the supernatant was observed at 96 hours p.i. (1.54 × 10^6^ pfu/well). These data correlated very well with cell death and demonstrated that GLV-1h68 can efficiently replicate in MTH52c cells.

In addition, the infectivity of GLV-1h68 was assessed and compared by virus titration in two pairs of cell lines (see in Supplementary Material available online at doi 10.1155/2010/736907). In both cases, GLV-1h68 formed plaques 70–200 times more efficiently in cancer cells than in normal cells, indicating that GLV-1h68 preferentially replicates in tumor cells.

### 3.2. Confirmation of Infection and Replication of GLV-1h68 in MTH52c Cells in Cell Culture and In Vivo through Virus-Mediated Protein Expression

**Figure 2:**
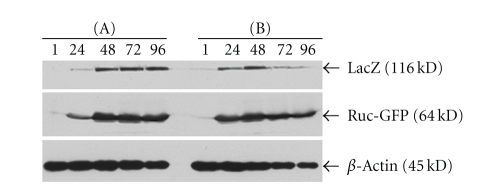
Western blot analysis of virus-mediated expression of Renilla luciferase-GFP fusion protein and *β*-galactosidase. MTH52c cells infected with GLV-1h68 at MOIs of 0.1 (a) and 1.0 (b) were used for protein isolation at 1, 12, 24, 48, 72, and 96 hours post infection (hpi). The time-dependent expression of Renilla luciferase-GFP fusion protein (Ruc-GFP), *β*-galactosidase (LacZ), and beta-actin as a control was analyzed as described in Materials and Methods.

To verify the infection and replication of GLV-1h68 in canine carcinoma cells, we followed the expression of the virus-mediated *Renilla* luciferase—green fluorescent protein—fusion protein (Ruc-GFP) and *β*-galactosidase (LacZ) in cell culture (Figure 2). Our Western blot analysis revealed that both marker proteins were efficiently expressed over a period of four days (Figure 2). The expression of Ruc-GFP and LacZ in cells infected at MOI of 0.1 peaked between 48 and 96 hpi, whereas maximum expression with an MOI of 1.0 was around 48 hpi. 

**Figure 3:**
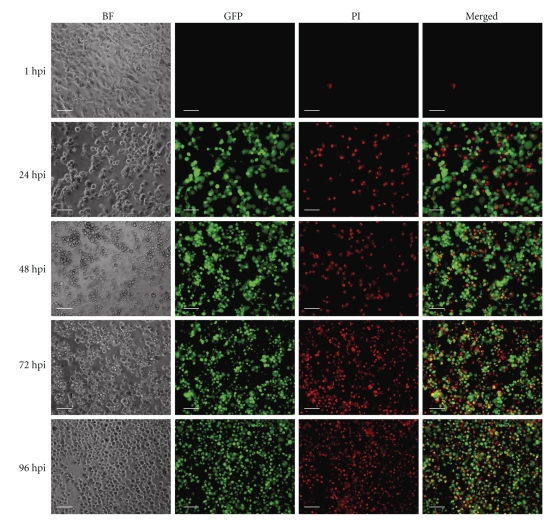
Time-dependent effects of infection of MTH52c with GLV-1h68 at an MOI of 1.0. (BF) Transmitted light view of virus-infected MTH52c cells; (GFP) expression of GFP in infected cells detected by direct fluorescence; (PI) propidium iodide staining of dead cells; (Merged) colocalization of GFP with the dead cells is shown in the merged imaged. All pictures in this set were taken at the same magnification. Scale bars represent 0.1 mm.

**Figure 4:**
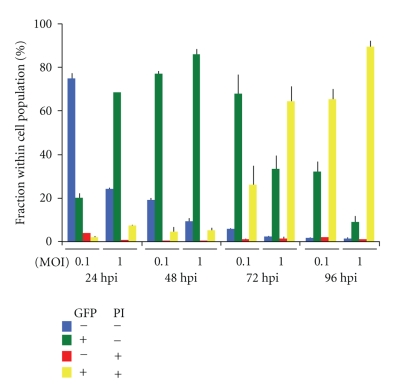
FACS analysis of MTH52c after infection with GLV-1h68 at MOIs of 0.1 and 1.0. Flow cytometry data indicate percentage of GFP and propidium iodide (PI) positive or negative cells.

Similar data were obtained by fluorescence microscopy (Figure 3). In these experimental settings we found that MTH52c cells infected with GLV-1h68 at MOI of 0.1 and 1.0 exhibited the strongest GFP expression at 72 and 96 hours, respectively, (supplementary Figure 1 and Figure 3). These data were also confirmed by flow cytometry (Figure 4) showing that the amount of infected cells increased over time and that those cells infected with vaccinia virus (detectable by GFP expression) were those cells that exhibited the major population of dead/dying cells (detectable by positive propidium iodide staining). In fluorescence microscopy, we used the same dye to demonstrate that most of the infected cells were dead/dying at 96h p.i. (Figure 3). These results indicate that GLV-1h68 was able to efficiently infect, replicate, and kill the MTH52c cells in cell culture. 

**Figure 5:**
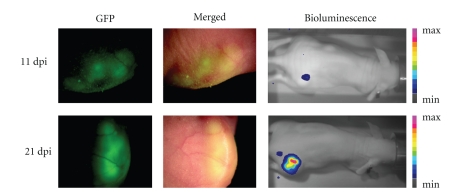
Fluorescence and luminescence imaging of MTH52c tumor-bearing mice after virus treatment. Fluorescence (GFP and Merged) imaging from the local tumor site and luminescence imaging of one representative mouse were taken 11 and 21 days post injection. Min: minimum; max: maximum.

Next, we examined the efficacy of GLV-1h68 to target MTH52c tumors *in vivo*. For this purpose, at different days postinjection, the mice of each group were observed either under a fluorescence stereomicroscope (Leica MZ 16 FA; Wetzlar, Germany) to detect GFP-dependent fluorescence or using the low-light Imager (NightOWL LB 981, Berthold Technologies, Bad Wildbad, Germany) to detect luciferase-catalyzed light emission in the presence of intravenously injected coelenterazine (Sigma, Taufkirchen, Germany). The GFP and luciferase expressions are dependent on vaccinia virus replication *in vivo*. As demonstrated, GFP fluorescence and luminescence were detected only within tumors of GLV-1h68-injected mice (Figure 5). The imaging data indicated the preferential accumulation of GLV-1h68 in MTH52c tumors.

### 3.3. A Single Systemic Application of GLV-1h68 Causes Significant Inhibition of Tumor Growth in MTH52c Xenografts

**Figure 6:**
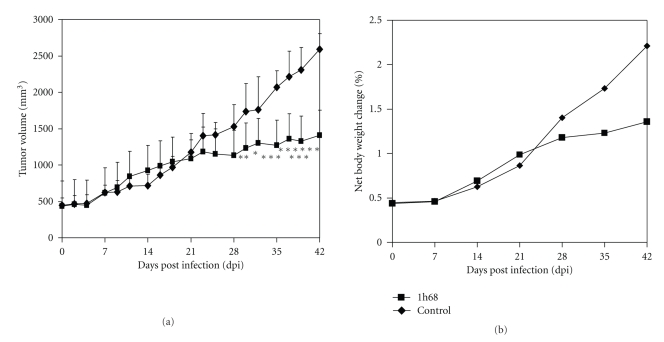
Effect of GLV-1h68 on MTH52c tumor growth in nude mice. (a) MTH52c tumor development in mice after GLV-1h68-treatment versus PBS treatment. Two-way analysis of variance (ANOVA) was used to compare the two corresponding data points of the two groups. *P* < .05 was considered as statistically significant *P < .05; **P < .01; ***P < .001. (b) Body weights of MTH52c cell xenografted mice after virus treatment.

The therapeutic capacity of GLV-1h68 against an induced canine mammary cancer was tested in 10 female nude mice implanted with MTH52c cells at the age of 6–8 weeks. Twelve days postimplantation, all nude mice developed tumors with sizes between 400 and 500 mm^3^. Then groups of five tumor-bearing mice were injected with either 5 × 10^6^ pfu of GLV-1h68 or PBS (control). The tumor size of all animals was measured thrice weekly for six weeks. The single vaccinia virus injection caused an efficient inhibition of tumor growth in all GLV-1h68-treated tumor-bearing mice compared to control mice (Figure 6(a)). In addition, no reduction of net body weight of the animals was observed (Figure 6(b)). 

The data revealed that GLV-1h68 could be an effective tool for the therapy of canine mammary carcinoma.

### 3.4. Viral Localization in GLV-1h68-Treated Mice after Inhibition of Tumor Growth

We analyzed the viral distribution in GLV-1h68-treated tumor-bearing mice by standard plaque assay and immunohistochemical staining. The plaque assay analysis revealed that viral titers in tumors were 10^4^ to 10^7^ and 10^3^ to 10^5^ times higher than the titers found in all the other organs combined at day 21 and 42, respectively (Table 1). These results show that GLV-1h68 can specifically infect and replicate in canine cancer cells.

**Figure 7:**
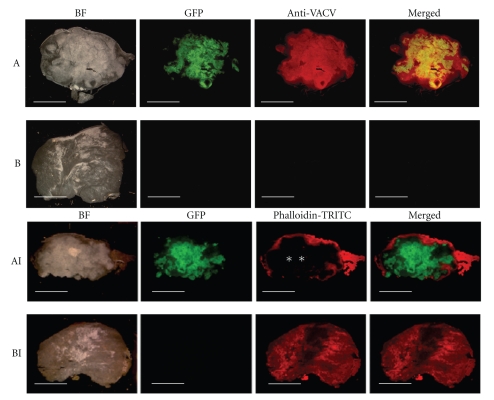
Immunohistochemical staining of MTH52c tumors. Tumor-bearing mice were i.v. injected either with 5 × 10_6_  pfu of At day 42 after injection, GLV-1h68 (A and AI) or PBS (B and BI). whole tumor cross-sections (100 *μ*m) were labeled either with antivaccinia virus or Phalloidin-TRITC (I) antibodies (both red) and analyzed by fluorescence microscopy to detect GFP (green) and actin or vaccinia virus-dependent (red) fluorescence. Scale bars represent 5 mm. *Large areas lacking actin staining indicate dead tumor tissue damaged by GLV-1h68.

To further examine the tumor tissues, we analyzed the tissue sections of the primary tumors at 42 days after virus injection by immunohistology. Microscopic analysis of viral distribution demonstrated that GLV-1h68 was present throughout the tumor tissue of virus-infected mice but not in control mice (Figure 7). As expected, both the virus and the GFP distribution were similar in the whole tumor tissue, indicating that in this case GFP expression is an optimal tool for the monitoring of the GLV-1h68 infection *in vivo* (Figure 7AB). In addition, the histological data revealed that vaccinia virus infection led to oncolysis and damage of tumor tissue (Figure 7AI).

### 3.5. Analysis of Host Immune Response in GLV-1h68-Infected and Noninfected Primary Tumors

In order to analyze the effects of virus infection *in vivo*, we determined the mouse antigen profiling of GLV-1h68-infected and noninfected tumors of MTH52c or ZMTH3 xenografted nude mice. Canine mammary ZMTH3 adenoma tumors are susceptible to GLV-1h68 treatment *in vivo *[12] and therefore were used as an additional control. 

At 42 days after virus injection, MTH52c and ZMTH3 tumors of nude mice with or without GLV-1h68 treatment were removed and used for generation of tumor tissue lysates as described in Material and Methods. 

The data in both xenograft models revealed that GLV-1h68 injection led to increased production of most of the tested proinflammatory cytokines and chemokines, such as MCP-1, MCP-3, MCP-5, M-CSF, IP-10, and IL-18 whereas only the cytokine MIP-1-gamma (CCL9) and the von Willebrand factor were downregulated (Table 2).

## 4. Discussion

Despite advances in surgery, radiation, and chemotherapy, the available treatment options for mammary carcinoma in dogs are limited and the prognosis for patients with advanced-stage disease is very poor. Therefore, the development of novel agents for therapy and diagnosis of canine mammary carcinoma is essential. 

In this study, we showed for the first time that the recombinant vaccinia virus GLV-1h68 was able to effectively infect, replicate in, and lyse canine carcinoma cells in culture. The viral replication correlated well with cell lysis and with expression of the marker GLV-1h68 genes encoding *β*-galactosidase and *Renilla* luciferase-green fluorescent protein (GFP) fusion protein, respectively. In addition, flow cytometry data (Figure 4) confirmed that the virus-infected cells, detectable by GFP expression, were those cells that exhibited the major population of dead/dying cells (detectable by positive propidium iodide staining). 

Taken together, we did not find any evidence of possible resistance of canine carcinoma cancer MTH52c cells to infection with vaccinia virus in cell culture. 

The current study also demonstrated the ability of GLV-1h68 to provide highly effective therapy *in vivo*. We observed a significant inhibition of tumor growth and damage of tumor tissue in the GLV-1h68-treated tumor-bearing mice compared to control mice. Most importantly, the treated animals appeared in good health without signs of toxicity, and no reduction of net body weight of virus-infected mice was observed (Figure 6(b)). In addition, experiments analyzing viral biodistribution in different organs (Table 1), as well as GFP fluorescence and luminescence studies on the living mice (Figure 5), confirmed the fact that the GLV-1h68 virus has an outstanding infection and replication capability and specificity in tumors [10, 13]. 

In order to analyze the possible mechanism of tumor elimination by GLV-1h68 in our MTH52c tumor xenograft model, we investigated the mouse immune-related protein antigen profiling in the primary tumors with or without virus injection. In these experimental settings we also used GLV-1h68-infected tumors of ZMTH3 xenografted nude mice as an additional control. The data revealed that in both the MTH52c and the ZMTH3 virus-infected tumors, the protein expression levels of most of the tested pro-inflammatory cytokines and chemokines were significantly upregulated compared to the corresponding noninfected tumors (Table 2). 

Many of the upregulated proteins, such as MCP-1, MCP-3, MCP-5, M-CSF, IP-10, and IL-18, augment innate immunity mediated by dendritic cells, neutrophils, macrophages, and NK cells. Interestingly, similar mouse immune-related protein antigen profilings were also determinated in other xenograft models after a single GLV-1h68 injection [13, 18]. Therefore, GLV-1h68 may induce upregulation of the innate immune system, leading to increasing levels of pro-inflammatory cytokines. This notion is also supported by recent immunohistological studies demonstrating specific peri- and intratumoral infiltration of MHC class II-expressing host cells (like e.g., macrophages, and dendritic cells) surrounding virus-infected cancer cells [18, 19]. The presence of activated macrophages or dendritic cells in virus-infected xenografts only could serve as an evidence for the association between xenograft eradication and activation of the innate immune system. These findings suggest that activation of the innate immune system may act together with viral oncolysis to induce inhibition of tumor growth and tumor eradication in this model. However, which components of the innate immune system are involved in the elimination of tumor cells remains unknown.

We have reported previously that the GLV-1h68 virus can be successfully used for the treatment of canine mammary ZMTH3 adenoma *in vivo* [12]. The comparison of the antitumor effects of GLV-1h68 with that of the present study showed that, in the adenoma xenograft ZMTH3 model, GLV-1h68 injection led to a faster and more efficient tumor inhibition and regression than in mice bearing MTH52c carcinoma tumors. One possible explanation could be the better replication efficacy of GLV-1h68 in the ZMTH3 tumors compared to that of MTH52c tumors *in vivo* [12]. However, a significant inhibition of the tumor growth was found in both ZMTH3 and MTH52c xenografts at day 30 after virus injection.

Therefore, GLV-1h68 could be a useful tool for treatment of both mammary cancer types in canine patients.

## 5. Conclusion

Our study demonstrates that the attenuated vaccinia virus strain GLV-1h68 can efficiently infect and destroy the canine mammary carcinoma MTH52c cells in cell culture and *in vivo*. In addition, a single systemic administration of GLV-1h68 causes a significant inhibition of tumor growth in MTH52c xenografts and damage of tumor tissue without detectable effects on the health status of the treated animals. 

In summary, these data indicate that GLV-1h68 is a promising candidate virus in the treatment of breast carcinomas in canine patients.

## Supplementary Material

Table 1: Comparison of the replication efficacy of vaccinia virus strains in cancer and in normal cell lines.

## Figures and Tables

**Table 1:** Viral titer in tissue samples (pfu/organ or tumor).

Animal no./dpi	1/21 dpi	2/21 dpi	3/21 dpi	4/42 dpi	5/42 dpi
Liver	120	1716	840	n.d	n.d
Lungs	1520	480	n.d.	56	324
Kidneys	n.d.	n.d.	n.d.	NT	NT
Spleen	150	n.d	n.d	n.d	n.d
Ovaries	n.d.	n.d	30	NT	NT
Tumor	2 × 10^7^	1 × 10^7^	2.1 × 10^7^	7.3 × 10^5^	7.3 × 10^5^

Tumor-bearing mice were injected with 5 × 10^6^ pfu of GLV-1h68. Mice were sacrificed at day 21 or 42 after virus injection (dpi). The data were determined by standard plaque assays on CV-1 cells using aliquots of the homogenized organs and were displayed as mean pfu/organ or tissue. For each organ, two aliquots of 0.1 ml were measured in triplicates.

n.d.: not detected (detection LIMIT<10 pfu/organ).

NT: not tested.

**Table:** (a) Protein expression level: upregulated (day 42 after virus infection).

Antigen	GLV-1h68/untreated ratio (MTH52c)	GLV-1h68/untreated ratio (ZMTH3)	Classification
Apo A1	7.56	1.21	Anti-inflammatory protein
IFN-gamma	4.00	1.8	Proinflammatory cytokine
IL-6	7.04	5.52	Proinflammatory cytokine
IL-11	6.59	1.6	Pleiotropic cytokine
IL-18	8.00	3.21	Proinflammatory cytokine
IP-10 (CXCL10)	29.47	11.21	Interferon-gamma-induced protein
MCP-1 (CCL2)	18.36	4,99	Proinflammatory cytokine
MCP-3 (CCL7)	13.26	2.36	Proinflammatory cytokine
MCP-5 (CCL12)	3.84	7.13	Proinflammatory cytokine
M-CSF (KC/GRO*α*)	8.28	3.51	Proinflammatory cytokine
MDC (CCL22)	4.02	1.97	chemokine
MIP-1beta	9.34	1.66	Proinflammatory cytokine
MIP-2 (CXCL2)	6.91	11.93	Proinflammatory chemokine
MMP-9	12.68	26.31	Matrix Metalloproteinase-9
TIMP-1	5.05	2.91	Tissue inhibitor of metalloproteinase type-1
TNF-alpha	6.47	1.5	Proinflammatory cytokine

**Table:** (b) Protein expression level: downregulated (day 42 after virus infection).

Antigen	untreated/GLV-1h68ratio (MTH52c)	untreated/GLV-1h68ratio (ZMTH3)	Classification
VWF	1.33	1.01	von Willebrand factor
MIP-1gamma (CCL9)	2.71	1.96	Macrophage inflammatory protein

## Abbreviations

ApoA1: Apolipoprotein A1
IL-6: Interleukin-6
IP-10: Interferon-inducible protein
MCP-1: Monocyte chemoattractant protein-1
M-CSF: Macrophage colony-stimulating factor
MIP: Macrophage inflammatory protein
MMP-9: Matrix metalloproteinase 9
MOI: Multiplicities of infection
TIMP-1: Metallopeptidase inhibitor 1
TNF-alpha: Tumor necrosis factor-alpha
VWF: Von Willebrand factor.
