# Supplementary material for: Significant Growth Inhibition of Canine Mammary Carcinoma Xenografts following Treatment with Oncolytic Vaccinia Virus GLV-1h68
Source: J Oncol. 2010 Jun 23;2010:736907. doi: 10.1155/2010/736907 (PMC2902752; doi:10.1155/2010/736907)
Supplement: Supplementary file 1 — Table 1: Comparison of the replication efficacy of vaccinia virus strains in cancer and in normal cell lines. [file 736907.f1.pdf]

Supplementary Material

Supplementary Table 1. Comparison of the replication efficacy of vaccinia virus strains in cancer and in normal cell lines.

The cell lines were treated with serial dilutions of viruses in duplicates. Two days post infection the cells were stained with 0.13% crystal violet. The viral titers in each cell line were calculated.

Tab.1A.: Virus infectivity in Hs578T vs. Hs578 Bst cells

**Virus**	**Titer (pfu/ml)**		
	human breast carcinoma cell line Hs578T [1]	human normal breast epithelial cell line Hs578 Bst [1] (from the same patient)	Hs578T versus Hs578 Bst
GLV-1h68	7.5×10^{8}	1.1×10^{7}	68.18
Lister strain (wild-type)	2.1×10^{9}	7.5×10^{8}	2.80
Western Reserve (wild-type)	2.1×10^{9}	2.1×10^{9}	1.00

Tab1B.: Infectivity of GLV-1h68 in hDF and HT-1080(pLEIN) cells [2]

**Cell line**	**Titer (pfu/ml)**
primary human dermal fibroblast (hDF) [2]	8.5×10^{6} +/- 1.4×10^{6}
human fibrosarcoma HT-1080 (pLEIN) [2]	1.7×10^{9}+/- 3.5×10^{6}

In both cases, GLV-1h68 formed plaques more efficiently in cancer cells than in normal cells, indicating GLV-1h68 preferentially replicates in tumor cells.

Literature:

1. Hackett AJ, Smith HS, Springer EL, et al. Two syngeneic cell lines from human breast tissue: the aneuploid mammary epithelial (Hs 578T) and the diploid myoepithelial (Hs 578Bst) cell lines. *J. Natl. Cancer Inst.* 1977; **58**: 1795-1806.

2. Patent application (WO/2008/100292) MODIFIED VACCINIA VIRUS STRAINS FOR USE IN DIAGNOSTIC AND THERAPEUTIC METHODS, Table 52, Example 33

Supplementary figure 1. Time-dependent effects of infection of MTH52c with GLV-1h68 at an MOI of 0.1

(**BF**) Transmitted light view of virus-infected MTH52c cells; (**GFP**) Expression of GFP in infected cells detected by direct fluorescence; (**PI**) Propidium iodide staining of dead cells and (**Merged**) co-localization of GFP with the dead cells is shown in the merged imaged. All pictures in this set were taken at the same magnification. Scale bars represent 0.1 mm.

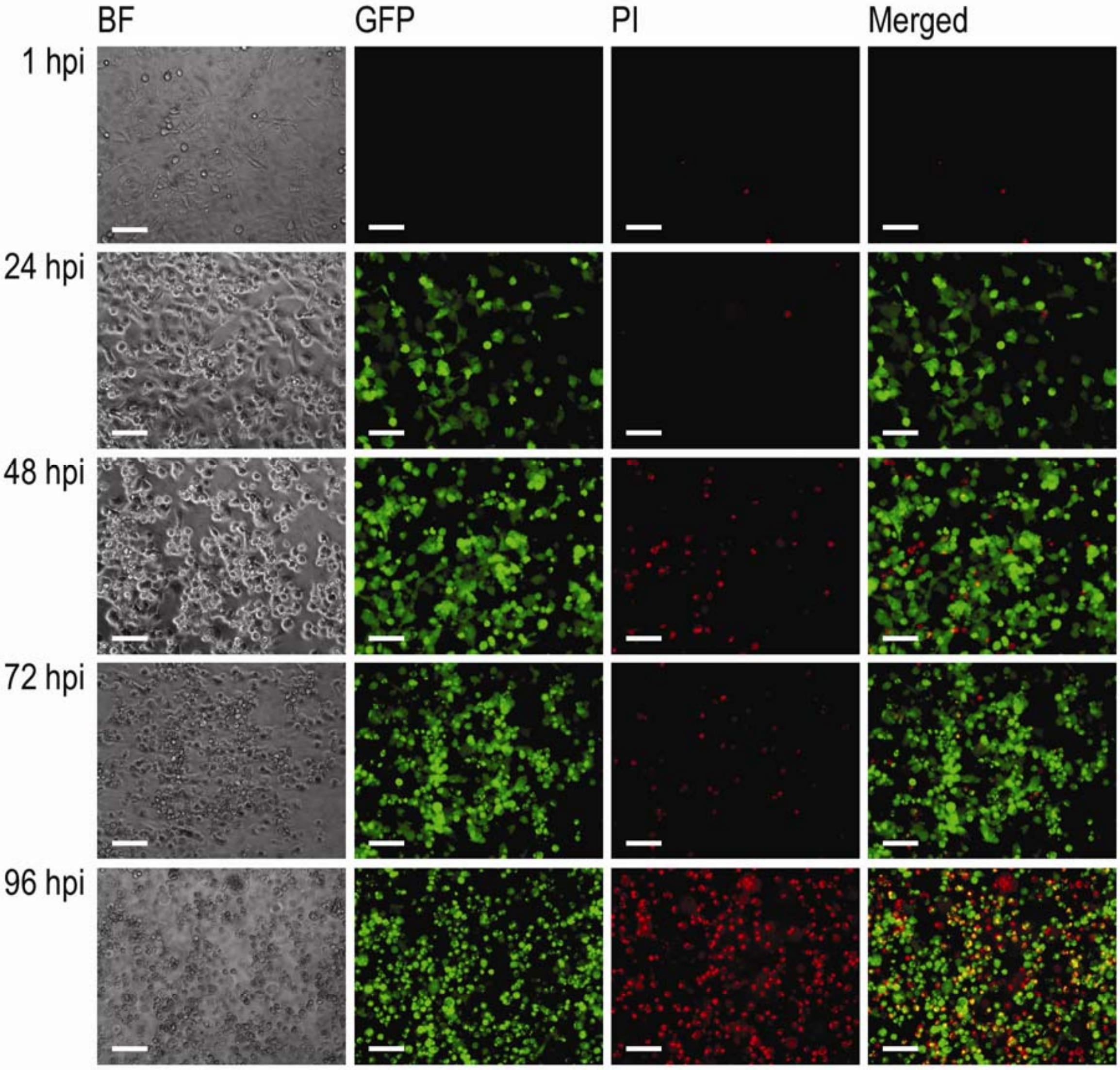
BF
GFP
PI
Merged
1 hpi
24 hpi
48 hpi
72 hpi
96 hpi
